# Romiplostim therapy as a second‐line treatment before splenectomy for refractory immune thrombocytopenia in a cirrhotic patient with iatrogenic Cushing syndrome secondary to corticosteroids

**DOI:** 10.1002/ccr3.667

**Published:** 2017-01-23

**Authors:** Teresa Casanovas Taltavull, Maria Carmen Peña‐Cala

**Affiliations:** ^1^ Gastroenterology ServiceHepatitis and Liver Transplant UnitIDIBELLHospital Universitari de BellvitgeFeixa Llarga s/nL'Hospitalet de Llobregat08907BarcelonaSpain

**Keywords:** Chronic liver disease, hypersplenism, iatrogenic Cushing syndrome, immune thrombocytopenia, romiplostim, splenectomy

## Abstract

Our case report discusses the usefulness of administering romiplostim as a second‐line treatment before splenectomy in a cirrhotic patient with immune thrombocytopenia who developed corticosteroid‐induced Cushing's syndrome. Corticosteroids were tapered and consequently withdrawn. The patient made a full recovery postsplenectomy.

## Introduction

This case report describes the clinical case of a patient with chronic liver disease (CLD) who suffered chronic immune thrombocytopenia (ITP), for more than one year which defines a chronic evolution.

Chronic ITP is an acquired immune disorder characterized by isolated thrombocytopenia. The diagnosis of ITP is established by the exclusion of other causes of low platelets count such as bone marrow failure, especially malignancy, infections, alcohol excess, or drug induced.

Severe clinical manifestations may be observed associated with ITP. Patients can suffer: gastrointestinal bleeding, extensive skin, mucosal, or intracranial hemorrhage. On physical examination, you can find splenomegaly and hepatomegaly. In our patient, these findings were attributed initially to his concomitant chronic liver disease. Our patient had compensated cirrhosis, Child A status, according to the prognostic classification of cirrhosis. His analytical parameters had only mild abnormalities severe; however, severe thrombocytopenia was observed which is not usually secondary to portal hypertension.

The patient received the first‐line treatments proposed by the clinical guidelines with intravenous immunoglobulins and prednisone. Despite showing no signs of improvement, the administration of prednisone was continued in the long term and the patient developed iatrogenic Cushing's syndrome. Because the patient failed to respond to first‐line treatment, a bone marrow examination was carried out. This test is usually not performed as an initial investigation if the history and clinical examination fit with the diagnosis of ITP.

The second line of recommended treatment is to perform a splenectomy, but the deteriorated clinical condition and comorbidities of our patient precluded it. At present, the recommendation for a second line of treatment is the administration of a thrombopoietin receptor agonists (TPO), romiplostim, or eltrombopag. In our patient, romiplostim was administered while tapering and suspending prednisone. When the patient was stabilized, splenectomy could be performed with the expected normalization of clinical situation and platelet count. Splenectomy is one of the second‐line treatments for adults with ITP.

## Case Presentation

In 2002, the patient, a 64‐year‐old male, presented at the emergency room with severe thrombocytopenia and a self‐limited intestinal hemorrhage of unknown origin and was diagnosed with chronic liver disease, Child A status. The cause of cirrhosis was attributed to alcohol consumption in the past. A bone marrow examination was performed and showed normal megakaryocytes, and as a result, hematological diseases were excluded. Thrombocytopenia persisted and was considered secondary to hypersplenism. The initial laboratory values are shown in Table 1, highlighting the severity of thrombocytopenia.

**Table 1:** Initial laboratory values

Laboratory tests	Value	Normal range
White blood cell count (cells/mm^3^)	3	3.9–10
Mean corpuscular volume (fL)	91	81–96
Platelets (cells/mm^3^)	**13**	**135–333**
Hemoglobin level (g/L)	120	126–166
Bone marrow examination	Showed no disorders	Not applicable
International normalized ratio (INR)	1.2	0.8–1.2
Creatinine (*μ*mol/L)	80	<111
Aspartate amino transferase (*μ*Kat/L)	0.51	<0.50
Alanine amino transferase (*μ*Kat/L)	0.38	<0.73
Alkaline phosphatase (*μ*Kat/L)	1.3	<1.5
Total bilirubin (mmol/L)	19	<18
Gamma‐glutamyl transpeptidase (*μ*Kat/L)	1.7	<1.11
Albumin (g/L)	38	33–50
Glucose (mmol/L)	5.6	4.1–6.9
Cobalamin/Folate (pmol/L/pmol/L)	291.9/6.8	≥122/>45.4
Lactate dehydrogenase (LDH) *μ*Kat/L	6.3	<3.4
Standard urinalysis	All within normal limits	Not applicable
Hepatitis B virus and hepatitis C virus Ab	Negative	Negative
Hepatitis autoimmune Ab (antinuclear, antimitochondrial, antismooth muscle, and anti‐KLM)	Negative	Negative

*μ*mol/L, micromoles per Liter; *μ*Kat/L, microkat per Liter; mmol/L, millimoles per Liter; pmol/L, picomol per Liter; Ab, antibodies.

Bold values means severity of low platelets count.

During the period 2005–2009, he suffered from repeated hemorrhages. Hence, he was treated with the first‐line therapy which is IV IgG and prednisone. During these years, he needed emergency treatments and was hospitalized on several occasions. In spite of standard therapies, hemorrhages persisted but he did not develop liver decompensation.

In June 2009, he was hospitalized for ecchymoses, rectal bleeding, and severe thrombocytopenia requiring blood transfusions, IV IgG, and high doses of prednisone (1 mg per kilogram of body weight per day). A bone marrow exam was repeated, and no abnormalities were detected.

In August, October, and November 2009, he needed to be re‐hospitalized for epistaxis and intestinal hemorrhage and his platelet count was 1.000–3.000 cells/mm^3^. Long‐term high doses of prednisone, 100 mg/day, were administered from June 2009 to April 2010. Hemorrhages were clinically stabilized, but the patient's clinical situation deteriorated.

In November 2009, he was referred to our clinic for evaluation of his chronic liver disease. He had developed iatrogenic Cushing syndrome with cardiovascular complications (shortness of breath, swollen extremities, weight increase). Table 2 shows the values of the patient's vital signs. General and specific analysis and abdominal doppler ultrasound were performed. Symptomatic treatment was established while tapering prednisone.

**Table 2:** Patient's vital sign values pretreatment with romiplostim

Blood pressure	180/90
Pulse (bpm)	67
Weight (Kg)/Height (cm)	102/168
BMI (Body Mass Index)	36.1

**Figure 1:**
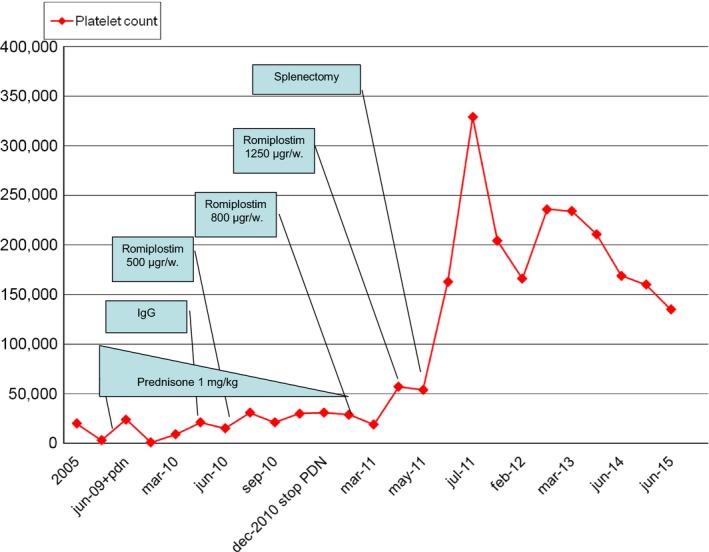
Platelet count level over time and correlation with medical treatment and splenectomy.

In March 2010, he started romiplostim treatment as a bridge for splenectomy. Taking into account his chronic liver disease and risk of vascular thrombosis, the target platelet count range was an average of 50,000–90,000 cells/mm^3^. Efficacy and safety were assessed weekly, during each visit. Figure 1 shows the platelet count during this period.

**Figure 2:**
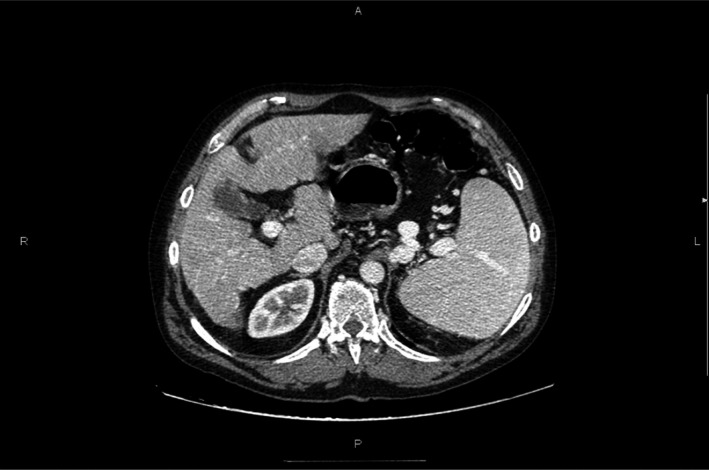
Abdominal CT scan lobular liver, suggesting cirrhosis without nparenchimal, biliary or vascular lesions, normal portal vein and enlarged spleen (19 cm.). Kidney of normal size and morphology. No retroperitoneal or lymphadenopathy and no free peritoneal fluid were observed.

In December 2010, prednisone was totally stopped and this allowed the patient to be considered for splenectomy. He was given an antipneumoccocal, meningococcal, and hemophilus influenzae vaccinations and underwent computed axial tomography (CAT) (Fig. 2) and abdominal ultrasound test. In addition, a nuclear medicine study was carried out of the liver–spleen image with 99 mTc‐labeled colloid, confirming enlarged spleen and a reduced platelet half‐life with a pattern of increased splenic destruction.

In May 2011, open laparotomy with splenectomy was performed with a liver biopsy specimen obtained preoperatively, which showed advanced fibrosis without steatosis or other features. After the surgery, the patient showed full recovery.

Today, after 5 years of follow‐up, the patient only requires vitamin supplements and standard ambulatory checkups every 6 months. His liver function remains stable, and platelet count is maintained at normal range.

## Discussion

We present the clinical case of a patient with chronic liver disease (CLD) and severe thrombocytopenia 1 who after 9 years of suffering repeated hemorrhages developed iatrogenic Cushing syndrome secondary to high doses of prednisone. He received romiplostim as a second line of immune thrombocytopenia (ITP) therapy 2 for a period of nine months while prednisone was tapered before a splenectomy could be indicated.

In CLD patients, thrombocytopenia is a common clinical problem associated with hypersplenism but ITP disorder is rarely encountered 1.

Thrombocytopenia in liver cirrhosis is typically less severe than that found in ITP, which may be secondary to hematological diseases, chemotherapy, some drugs, hepatitis C‐related, and myelodysplastic syndromes 3. In our patient, secondary causes of ITP were ruled out and the diagnosis of ITP was well established.

Treatment of ITP in our patient was a challenge due to the risks associated with liver disease and also to the Cushing Syndrome and cardiovascular complications observed 4. Therefore, our major concern while decreasing the doses of prednisone was how to treat the underlying thrombocytopenia and hypercortisolism with heart failure and hypertension avoiding steroid withdrawal syndrome.

Nowadays, clinical guidelines for ITP in newly diagnosed adults recommend rituximab, thrombopoietin receptor agonist (TPO), TPO‐RA (romiplostim or eltrombopag), or splenectomy for patients who are unresponsive or who have relapsed after initial IV IgG and corticosteroid therapies. Splenectomy is one of the second‐line treatment for adults with ITP. 5, 6.

It is important to note that in case of resistance to the first‐line therapy, as was observed in our patient, other options such as splenectomy should be considered. However, in patients with CLD, portal hypertension and thrombocytopenia represent obstacles to surgical procedures.

Splenectomy has been the standard second‐line treatment for adults with ITP and remains a good option 7. Due to the deleterious effects of long‐term use of high doses of prednisone, our patient's clinical condition was severely deteriorated, and this was a cause for deterring and contraindicating the splenectomy that should have been performed once clinicians realized that the patient was nonresponding to the first‐line treatment. Doctors may be hesitant to indicate splenectomy as some adults improve over time or spontaneously or as a result of medical treatment.

Avoiding or delaying splenectomy is usual and is evidenced by a decrease in the rate of splenectomies performed in recent years.

However, the majority of groups accept splenectomy as a second‐line treatment after failure of steroids and in the absence of contraindication. Failure is defined by a remaining platelet count of less than 10,000–30,000, active bleeding, or high steroid requirement 8.

Although romiplostim had not been tested specifically in chronic liver disease, after examining the pros and cons, it was indicated.

Many recent clinical case reports have shown a possible role of romiplostim in the preparation preliver transplant or prior for invasive therapeutic procedure 9, 10, 11.

Thrombocytopenia associated with advanced liver disease has a multifactorial origin. Usually a combination of causes can be implicated, producing an imbalance between production and destruction of platelets. A reduction of synthesis of TPO, which is produced exclusively by the liver, has been observed and also an increased splenic sequestration as a consequence of portal hypertension 12.

The etiology of thrombocytopenia in our patient was attributed to hypersplenism, although since the beginning a component of ITP could not be discarded. Despite performing a scintigraphic evaluation which confirmed the platelet sequestration and destruction in the spleen, these results are not totally predictive 13. Complications related to romiplostim treatment have been described: thrombotic events related to an elevated number of platelets, lack of response due to fibrous tissue deposition in bone marrow, worsening thrombocytopenia due to a rebound effect etc. 14.

It is worthwhile presenting this patient because not only did treatment with romiplostim improves platelet count but it also enabled a clinical improvement, thus allowing him to be splenectomised 15.

Due to the presence of iatrogenic Cushing syndrome with cardiovascular complications in our patient, there were some concerns regarding the impact of romiplostim on his management. Nevertheless, romiplostim was well tolerated. However, the possibility of severe complications, especially in patients with decompensated cirrhosis, should be considered.

The outcome after splenectomy was uneventful. Cirrhosis was not decompensated during the treatment period, and a Child A status was maintained indicating a relatively good liver function and a good prognosis for surgery.

## Conclusion

This case report emphasizes the hazards of long‐term administration of prednisone, which in our patient resulted in Cushing's syndrome, and shows how romiplostim was used to maintain a safe platelet count while tapering prednisone to prepare the patient for splenectomy.

## Conflict of Interest

None declared.
